# Long-Term Evaluation of Poly(lactic acid) (PLA) Implants in a Horse: An Experimental Pilot Study

**DOI:** 10.3390/molecules26237224

**Published:** 2021-11-29

**Authors:** Júlia Ribeiro Garcia Carvalho, Gabriel Conde, Marina Lansarini Antonioli, Clarissa Helena Santana, Thayssa Oliveira Littiere, Paula Patrocínio Dias, Marcelo Aparecido Chinelatto, Paulo Aléscio Canola, Fernando José. Zara, Guilherme Camargo Ferraz

**Affiliations:** 1 School of Agricultural and Veterinarian Sciences—FCAV, São Paulo State University—UNESP, 14884-900 Jaboticabal, São Paulo, Brazil; juliargc@hotmail.com (J.R.G.C.); gabriel.conde@unesp.br (G.C.); m.antonioli@unesp.br (M.L.A.); t.littiere@unesp.br (T.O.L.); paulo.canola@unesp.br (P.A.C.); fjzara@fcav.unesp.br (F.J.Z.); 2 Veterinary School, Federal University of Minas Gerais—UFMG, 31270-901 Belo Horizonte, Minas Gerais, Brazil; santana.chs@gmail.com; 3 São Carlos School of Engineering—EESC, University of São Paulo—USP, 13566-590 São Carlos, São Paulo, Brazil; pauladpdias@usp.br (P.P.D.); mchinelatto@sc.usp.br (M.A.C.)

**Keywords:** biocompatibility, biodegradation, biomaterial, polylactide-based materials, polymer, scanning electron microscopy

## Abstract

In horses, there is an increasing interest in developing long-lasting drug formulations, with biopolymers as viable carrier alternatives in addition to their use as scaffolds, suture threads, screws, pins, and plates for orthopedic surgeries. This communication focuses on the prolonged biocompatibility and biodegradation of PLA, prepared by hot pressing at 180 °C. Six samples were implanted subcutaneously on the lateral surface of the neck of one horse. The polymers remained implanted for 24 to 57 weeks. Physical examination, plasma fibrinogen, and the mechanical nociceptive threshold (MNT) were performed. After 24, 28, 34, 38, and 57 weeks, the materials were removed for histochemical analysis using hematoxylin-eosin and scanning electron microscopy (SEM). There were no essential clinical changes. MNT decreased after the implantation procedure, returning to normal after 48 h. A foreign body response was observed by histopathologic evaluation up to 38 weeks. At 57 weeks, no polymer or fibrotic capsules were identified. SEM showed surface roughness suggesting a biodegradation process, with an increase in the median pore diameter. As in the histopathological evaluation, it was not possible to detect the polymer 57 weeks after implantation. PLA showed biocompatible degradation and these findings may contribute to future research in the biomedical area.

## 1. Introduction

Biodegradable materials have been widely studied for medical applications during the last decades due to their numerous benefits over non-biodegradable materials. Importantly, since these materials disappear after degradation, the implants do not need to be removed. Furthermore, implants prepared using biodegradable polymers can prevent recurrences in the event of fractures, as they can be designed to degrade at a rate that will slowly transfer the load to the healing bone, thus allowing for adequate healing [1,2]. However, these materials also have some technical limitations that make them difficult to process and use as a final product, such as limited control over physicochemical properties, because of the intrinsic raw material variability and difficulties in adjusting degradation rates [1,3].

Poly(lactic acid) (PLA) is one of the most widely used biopolymers and has the potential to be used as an alternative to high-cost, non-biodegradable biocompatible materials. PLA holds promising applications in several areas, particularly in packaging, agricultural products, disposable materials, and the biomedical industry [4,5]. Among its advantages we list its biodegradability, renewability and low environmental impact when discarded. In addition, PLA has good biological safety, mechanical resistance, and processability profiles [6,7]. In the biomedical area, PLA has been used as a degradable suture, bone fixation device, material for surgical implants, drug delivery systems, and scaffolds for human and rodent tissue engineering applications [1,6,8,9].

Therefore, the medical application of PLA in horses can be an exciting opportunity for the experimental development of new biomaterials, as this species undergoes a fast and excessive healing process, which tends to follow abnormal repair reactions such as the formation of exuberant granulation tissue [10,11]. Moreover, with a global herd totaling about 61 million heads and a relevant world market, this animal species is part of the cattle production chain, utilized as a companion animal or in numerous sports, with emphasis on Olympic sports [12,13]. Thus, PLA may be an interesting alternative with potential application in equine medicine.

In the equine species, therapeutic polymer implants can mimic tissues, promote cell proliferation, and tissue reconstitution, forming a conjunctive capsule. Moreover, they can be combined with drugs for systemic or local action. However, polymer implants must be biocompatible and bioabsorbable [4,8]. There are few studies on the use of polymer implants in horses, with most of them involving their use to repair bone fractures [13,14,15,16,17]. Therefore, polymeric materials represent potential alternatives to the use of autologous and heterologous grafts, which have been limited to fracture correction in equine medicine [13,15]. Furthermore, biomaterials have been used for the treatment of joint damage [18,19,20] and drug delivery [21]. Among these studies, only one used PLA for internal fixation of fractures of horses’ proximal sesamoid bone. PLA proved superior to metallic implants because the animals presented a lower degree of lameness and better-quality bone remodeling [14].

Our research group recently evaluated the biocompatibility and biodegradability of PLA and a polymer blend based on PLA and poly(ε-caprolactone) (PCL) compatibilized with a copolymer derived from ε-caprolactone and tetrahydrofuran, which was implanted subcutaneously in horses. However, the materials were only implanted for 24 weeks [22]. Herein, we expand these findings by evaluating the biocompatibility and biodegradability of six PLA implants in a horse up to 57 weeks. Such long-term evaluation of polymer implants has not been performed previously in horses. Our data shows that the PLA implants do not provoke toxic reactions and can be applied safely in vivo for extended times in the equine species.

## 2. Results

### 2.1. Clinical Evaluation

Clinical evaluation was carried out during the experiment to verify the health evolution of the horse and/or the possible presence of systemic changes induced by the implantation of the biomaterials. In general, there was no discomfort associated with the implantation of the materials, and no behavioral and/or appetite changes were observed. Intestinal motility, hydration status, apparent mucous color, and capillary filling time were within the normal range for the species. RR and RT values were within reference values for adult horses throughout the implantation period (8–16 mpm; 37–38.3 °C; [23]), (Figure S1b,c). HR values increased at times up to 144 h after implantation; however, they returned to normal values for the equine species after 168 h (28–44 bpm; [23]) (Figure S1a).

### 2.2. Plasma Fibrinogen

PF concentration was determined to check for systemic inflammatory processes due to the implantation of the biopolymers. PF values remained within the range of reference for the equine species (100–400 mg/dL; [24]) throughout the evaluation period (Figure S2).

### 2.3. Mechanical Nociceptive Threshold (MNT)

**Figure 1:**
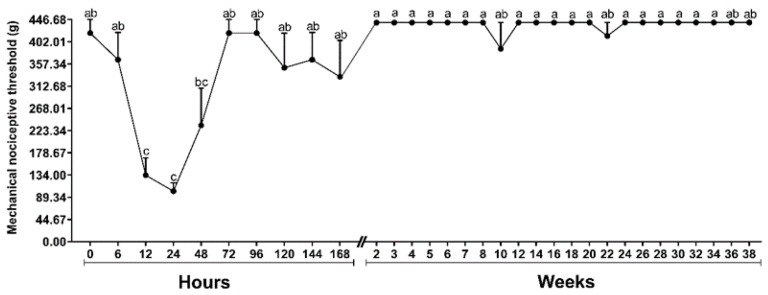
Graphic representation of the means ± standard error of the mechanical nociceptive threshold (MNT) of a horse submitted to the implantation of six polymers of poly(lactic acid) (PLA). Means followed by the same letter do not differ by the Tukey’s test (*p* < 0.05) in each moment.

Compared to baseline, MNT reduced 12 and 24 h (*F* = 9.431; *p* ≤ 0.001) after implantation. Forty-eight hours after implantation MNT was reversed (Figure 1).

### 2.4. Histopathological Analysis

**Figure 2:**
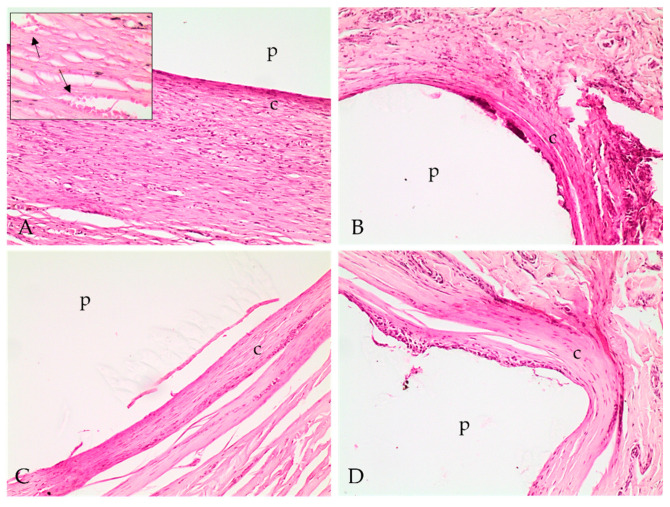
Photomicrographs of implant polymer sites with the polymer (p) and fibrotic capsular formation (c) involving the material. (**A**) Fibrotic capsular formation at 24 weeks; (**B**) 28 weeks; (**C**) 34 weeks; and (**D**) 38 weeks. Hematoxylin and eosin stain, 100×. (A) Inlet: fibrotic capsular formation at 24 weeks with small vases proliferation (arrows), characterizing angioplasia. Hematoxylin and eosin stain, 400×.

**Figure 3:**
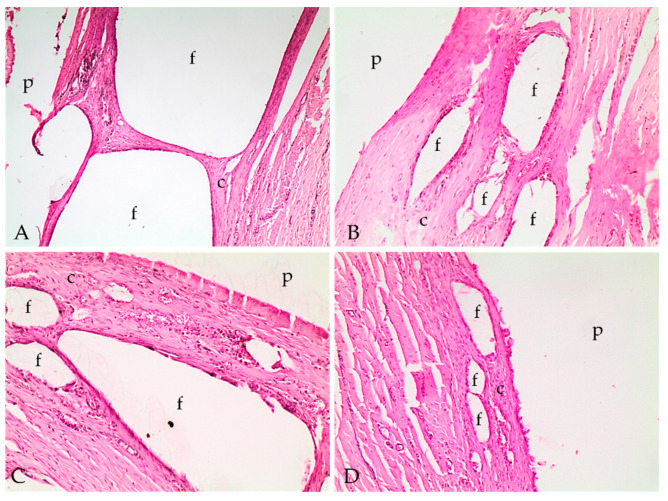
Photomicrographs of implant polymer sites with the capsular formation (c), polymer (p) and cellular growth with fragmentation of the polymer (f). (**A**) Cellular growth and polymer fragments at 24 weeks; (**B**) 28 weeks; (**C**) 34 weeks; and (**D**) 38 weeks. Hematoxylin and eosin stain; 40× (**A**) and 100× (**B**–**D**).

**Figure 4:**
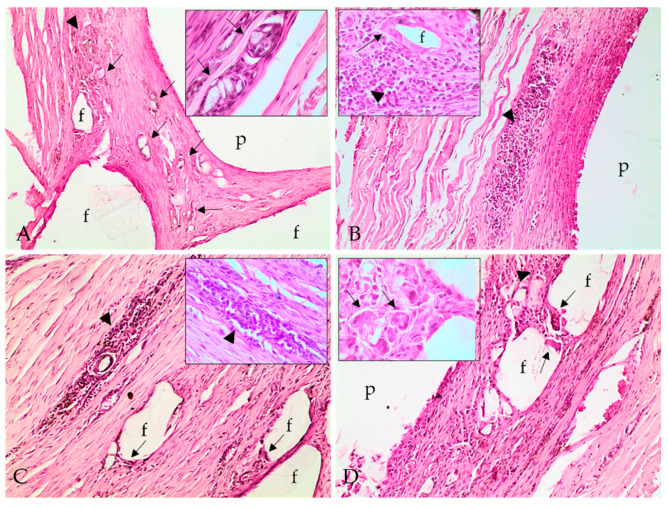
Photomicrographs of implant polymer sites with the polymer (p), lymphoplasmacytic inflammatory infiltrate (arrowhead), and epithelioid macrophages and multinucleated giant cells, with intracytoplasmic polymer material, thus characterizing polymer phagocytosis (arrows). (**A**) Inflammatory infiltrate and polymer phagocytosis at 24 weeks; (**B**) 28 weeks; (**C**) 34 weeks; and (**D**) 38 weeks. Hematoxylin and eosin stain; 100×. Inlet (**A**,**D**): multinucleated giant cells with intracytoplasmic fragments of polymer material, characterizing phagocytosis (arrows). Inlet (**B**,**C**): lymphoplasmacytic inflammatory infiltrate (arrowhead). Inlet (**C**): epithelioid macrophages (arrow) delimiting a polymer fragment (f). Hematoxylin and eosin stain; 400×.

**Figure 5:**
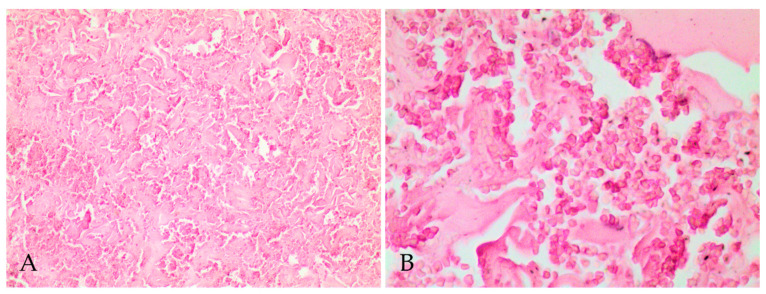
Photomicrographs of implant polymer site removed 57 weeks after implantation. No polymer was identified at this time (**A**) Site of implantation with abundant disorganized collagen fibers, no integral or fragmented polymer material or fibrotic capsule and intense diffuse hemorrhage. (**B**) Site of implantation evidencing the intense diffuse hemorrhage, characterized by many erythrocytes out of vessels. Hematoxylin and eosin; 100× (**A**) and 400× (**B**).

Histopathologic evaluation revealed fibrotic capsular formation involving the polymer at all implants surgical removal times (Figure 2), except at 57 weeks; however, over time, the capsules became more organized, as indicated by the progression in the scores of capsule characterizations (Table 1). We observed cellular growth, characterized as fragmentation of the biomaterial and the invasion of the fibrotic tissue, and the consequent surrounding of the evaluated fragments, which got smaller at late implant removal times (Figure 3 and Table 1). We also detected lymphoplasmacytic and histiocytic inflammatory infiltrates associated with the fibrotic capsule, including epithelioid macrophages and giant multinucleated cells with intracytoplasmic polymer fragments that increased with time (Figure 4 and Table 1). These features revealed phagocytosis of the polymer, which was more evident at late implant removal times (Table 1). Further, the fibrotic capsule of all implants showed angioplasia (Figure 2). The sample removed after 57 weeks of implantation presented hemorrhage, some neutrophils, moderate angioplasia, and an abundant presence of collagen fibers. However, no polymer material or fibrotic capsule were identified, hampering the scoring of the fragmented tissue (Figure 5).

### 2.5. Scanning Electron Microscopy (SEM)

**Figure 6:**
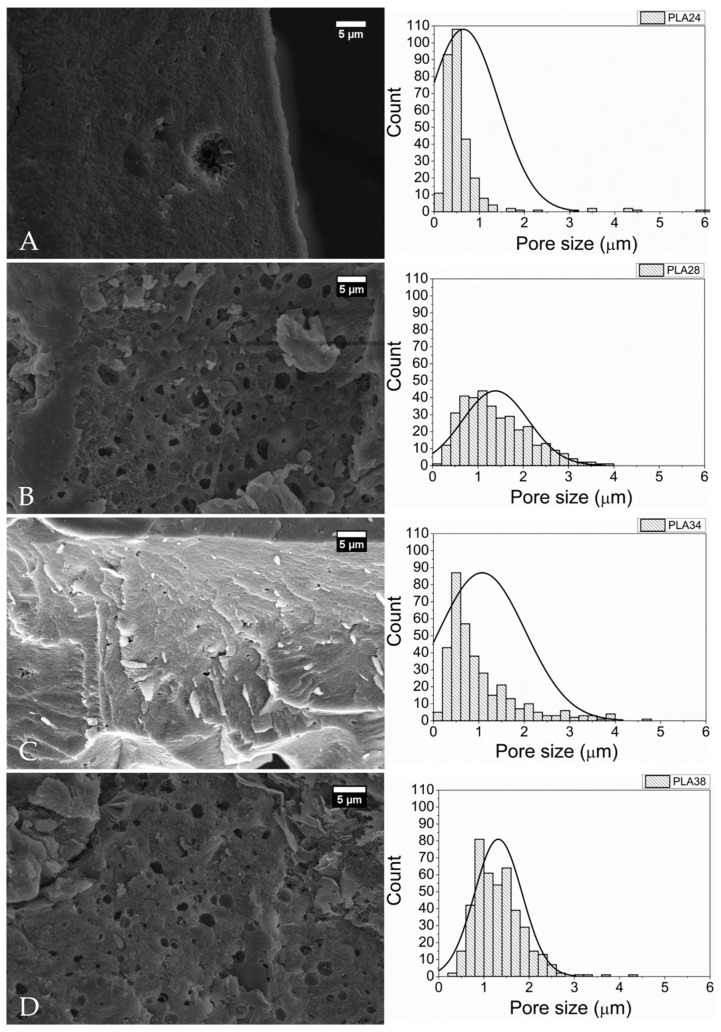
SEM micrographs and pore size distribution histograms of the surface of PLA implanted subcutaneously in one horse. (**A**) Twenty-fourweeks following implantation; (**B**) 28 weeks following implantation; (**C**) 34 weeks following implantation; and (**D**) 38 weeks following implantation.

**Figure 7:**
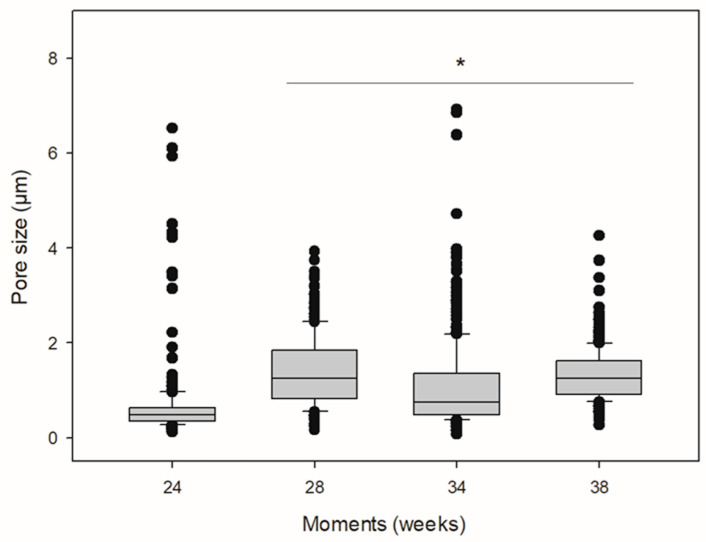
Medians and amplitude of pore size of PLA implanted subcutaneously in one horse. * Indicates increase in pore size when compared to pore size at 24 weeks following implantation (Kruskal-Wallis, *H* = 423.113; *p* < 0.001).

Figure 6 reveals SEM micrographs of PLA surfaces implanted and exposed to biodegradation for 24 (Figure 6a), 28 (Figure 6b), 34 (Figure 6c), and 38 weeks (Figure 6d) and their respective pore size distribution histograms. The distribution of pore diameters was not normal. The median pore size is shown in Table 2. The data revealed surface irregularity on the surface, porous morphological appearance, and the presence of cracks at all times, indicating a process of biodegradation on the material. Moreover, there was an increase in pore diameter at all times compared to the first moment (24 weeks) (Kruskal-Wallis, *H* = 423.113; *p* ≤ 0.001) (Table 2 and Figure 7).

**Figure 8:**
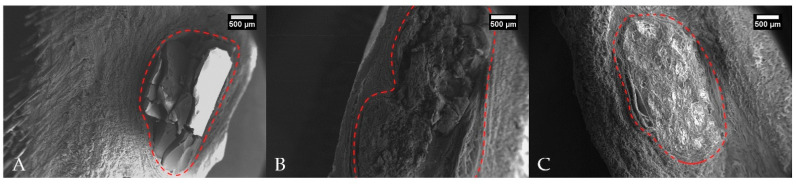
SEM micrographs of skin fragments with PLA implanted in one horse (**A**) 34 weeks following implantation; (**B**) 38 weeks following implantation; and (**C**) 57 weeks following implantation. Dotted red lines delimits the area of the implants.

Furthermore, over time, the polymer underwent degradation and changes in its morphology up to 57 weeks. Afterward it was no longer possible to detect the polymer within the skin fragment, thus impeding the removal of polymer fragments for analysis without the skin (Figure 8). We must recognize a limitation of our study. Owning to logistic restrictions, we have not been able to produce aa SEM image from a non-implanted PLA sample. However, this limitation does not compromise the study findings and more information on this non-implanted sample can be found in Carvalho et al., 2020 [22].

## 3. Discussion

We previously studied the changes related to the safe biodegradation of the PLA polymer implanted subcutaneously in horses for 24 weeks [22]. Here we evaluated the changes incurred in a horse over 57 weeks.

We found that PLA implantation did not induce systemic inflammatory responses in the horse studied. HR values increased up to 144 h after implantation. This may have occurred because there was more frequent handling of the animal in this evaluation period, which probably triggered a mild anticipatory sympathetic response to general management. Indeed, HR decreased from the seventh day of implantation, remaining within normal values for equine species. Likewise, the concentrations of plasma fibrinogen, an acute-phase protein commonly used to diagnose and monitor various inflammatory conditions in equine medicine [25], revealed that the injury caused by the skin incisions and the implantation of the polymers was not able to elicit a systemic inflammatory response.

On the other hand, MNT evaluation revealed the presence of a local inflammatory response, characterized by a decrease in nociception. Von Frey Filaments, used to assess the skin MNT, measures cutaneous hyperalgesia and allodynia, and usefully mimics clinical conditions that present increased cutaneous sensitivity [26]. In a study that evaluated the preemptive analgesic effect of epidural ketamine before performing skin incisions with sutures in horses, VFF were also able to quantify skin sensitivity [27].

Histopathological analysis revealed the formation of a fibrotic capsule delimiting the biomaterial from the initial stage of the inflammatory response, remaining present until 38 weeks post-implantation. Encapsulation of biomaterials occurs due to a chronic foreign body reaction [28]. In comparison with PLA24, the PLA38 presented a fibrotic capsule with multiple layers of fibroblasts and essential changes. Among these changes, we detected severe cell growth of fibrous tissue and polymer fragmentation, marked lymphohistioplasmocytic inflammation, marked phagocytosis of the material, and moderate angioplasia, which are characteristic alterations of a chronic inflammatory response [29].

Inflammation around the material, characterized by the presence mainly of lymphocytes and macrophages, accompanied the cell growth of the fibrous tissue amidst the biopolymers, which represented the local chronic inflammation and organization of this tissue. The presence of macrophages in the capsule and at the material’s interface was observed in other studies, being related to phagocytosis and implant clearance [8,30]. Interestingly, accentuated phagocytosis was observed in both PLA24 and PLA38 samples. The intense phagocytosis observed in PLA38 was possibly related to an intense fragmentation and biodegradation of the biomaterial, which incited macrophages to phagocytose the resulting fragments for tissue cleaning. Fragmentation of the material leads to cell growth, and the resulting capsule insulated the material while some fibrous tissue infiltrated the polymer, thus promoting further fragmentation.

The proliferation of fibrous tissue around polymer fragments may be associated with the presence of pores and cracks in the materials, as observed by SEM. These pores promote cell growth as they provide a greater surface area [31]. Fifty-seven weeks after implantation, the polymer was no longer detected within the skin fragment. The assessment interval between 38 and 57 weeks is significant and relatively large, and it is not possible to determine what occurred in this period. However, a progression on polymer fragmentation associated with tissue clearance possibly happened to the point when the material was no longer detected. Thus, the fibrotic capsule was replaced by fibrovascular tissue rich in organized and well-differentiated collagen, similar to the collagen observed deep in the dermis. We also speculate that the intense phagocytosis observed in one of the polymers at 24 weeks following implantation is probably related to an initial foreign body response.

In addition to macrophages and multinucleated giant cells, lymphocytes were often present in the infiltrates around the implants. These findings are probably associated with a chronic inflammatory response at the injury site, which is usually characterized by the presence of macrophages, multinucleated giant cells, monocytes, and lymphocytes, in addition to fibrosis and angioplasia [32]. Furthermore, angioplasia was observed at all evaluated times, with mild intensity in PLA24 and moderate intensity in the other materials. The process of well-differentiated vascular proliferation is essential for tissue nutrition, maintenance of cell proliferation, and migration of inflammatory cells [8].

SEM data, obtained from the sample removed 24 weeks following implantation, revealed signs of biodegradation of the PLA. However, few pores were present with an median diameter of 0.477 μm. As post-implantation time progressed, we observed a gradual increase in the number of surface pores and a large variation in pore size. In vivo biodegradation of PLA depends on the intrinsic characteristics of the material, such as the D-PLA content, crystallinity, and molar mass and the recipient’s conditions such as temperature, pH, and presence of cellular infiltrates [33]. The PLA used herein has a low content of D-PLA, which hampers its biodegradation. Indeed, chains with a predominance of L-PLA can organize into crystalline structures that are less susceptible to the permeation of water and extracellular enzyme infiltrates [34]. The biodegradation time established for the low content D-PLA implant used herein can be considered high when compared to that reported by Tschakaloff et al. (1994) [35], which showed that 70% of the mass of a L,D-PLA implant in rabbits biodegraded within 14 days.

Another important factor is the size of the PLA chains since only small molecules can enter cells and be used for cell metabolism. The average molar mass of the PLA used in this study is high (around 80,000 g.mol^−1^). The main in vivo biodegradation mechanism of PLA is the hydrolysis of ester groups, which leads to a slow reduction in chain size by chain scission to small oligomers or unitary lactic acid molecules. Once reduced to small lactic acid oligomers, they can be phagocytosed and take part in cellular metabolism [33].

The distribution of the histograms revealed that the biodegradation pattern of the polymer occurs with the emergence of small pores on the material’s surface promoted by the hydrolysis and breakage of polymer chains by extracellular enzymes [33]. Small superficial pores allow the infiltration of water and extracellular enzymes into the polymeric bulk, thus promoting the appearance of more small pores inside the material. Eventually, the chains between the small pores are consumed, and the pores become larger. For this reason, the median pore size was very similar between samples with different post-implantation times, but the analysis of the micrographs revealed a higher number of pores as the post-implantation time progressed until the polymer was completely biodegraded.

The present in vivo study evaluating the long-term biocompatibility and biodegradability of PLA implants in a horse is innovative and suggest that this biopolymer can be safely used in equine medicine. However, PLA has low flexibility and resistance, high apolarity, and low degradation rate, which are limiting properties for its use in human and veterinary medicine. Several approaches could be employed to overcome these limitations or to adjust the physical properties of the currently available PLA, such as the production of polymeric blends. Interestingly, although the PLA used herein was not of medical grade, it proved to be biocompatible and provided encouraging results that could pave the way for new low-cost biomaterials. In any case, future studies addressing the effectiveness of PLA for further uses, such as drug development, tissue engineering, equine surgery, and general medicine are warranted.

## 4. Materials and Methods

### 4.1. Ethics Statement

The study followed the Ethical Principles in Animal Experimentation adopted by the Brazilian College of Animal Experimentation and was approved by the Ethics Committee on Animal Use of the CEUA–FCAV/Universidade Estadual Paulista (UNESP) under protocol n° 006548/17.

### 4.2. Material Preparation

The polymer was prepared at the Department of Materials Engineering at the São Carlos School of Engineering, University of São Paulo, Brazil, according to Dias e Chinelatto (2019) [36]. The material used was Poly(acid lactic) (PLA grade: Ingeo 3251D), manufactured by NatureWorks Co. Ltd. with 80,000 g.mol^−1^, 1.4% of D-PLA content, 48 MPa tensile strength, 2.5% strain at break, and 16 J.m^−1^ Izod impact resistance. Polymer implants (1 cm^2^ and 1 mm thick) were produced via hot pressing at 180 °C. Sterilization was performed with ethylene oxide (Acecil Central de Esterilização Comércio e Indústria Ltda, Campinas, São Paulo, Brazil).

### 4.3. Animals

We used one adult horse, gelding, crossbreed, weighing 350 kg, 15 years old, from the didactic herd of the Laboratory of Equine Exercise Physiology and Pharmacology (LAFEQ/DMFA/FCAV), UNESP. The animal was kept in a paddock, fed with corn silage, water, and mineral salt ad libitum, in addition to 0.2% of body weight of a mash feed once a day. Before study commencement, the horse underwent a complete physical examination, in addition to hematological and biochemical tests, to determine its health status. The horse was previously treated with anthelmintic (repeated every four months) and vaccinated against rabies, tetanus toxoid, east and west equine encephalomyelitis, and equine influenza types A1 and A2.

### 4.4. Polymers Implantation

**Figure 9:**
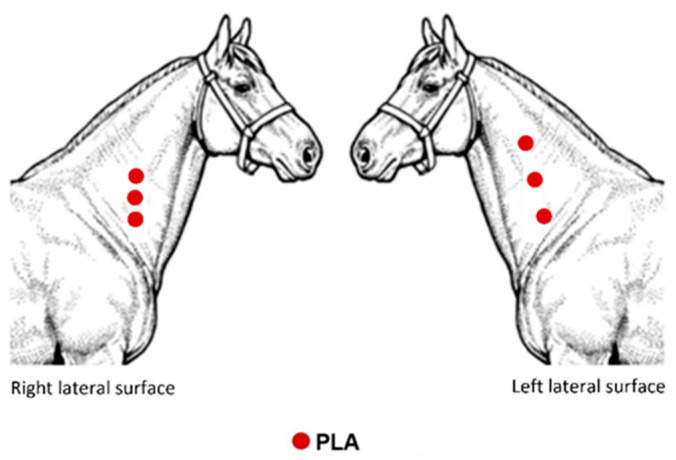
Schematic illustration showing the evaluation sites of a horse implanted with PLA.

An overview of the implantation sites is depicted in Figure 9. The lateral surface (LS) of the neck was used as the implant site. The animal evaluated here was used in the pilot study that preceded our previous research [22]. The animal received the PLA implant in different locations between the right and left LS, aiming to investigate whether there would be interference from the inflammatory process between the implants, and whether the difference in thickness of the subcutaneous tissue observed via ultrasound would interfere with the inflammatory process.

Before implanting the biomaterial, six areas of approximately 8 cm2 on the LS (three on the left and three on the right) were shaved, and antisepsis was performed with 2% chlorhexidine digluconate and 70% ethyl alcohol. Subsequently, the animal was sedated with intravenous administration of xylazine hydrochloride (1 mg.kg^−1^). Afterward, local anesthetic infiltration was performed around each incision site with 2.0 mL of 2% lidocaine hydrochloride. A 2 cm horizontal incision was made using a #15 scalpel blade in the determined area on the LS. A space between the skin and cutaneous muscle was obtained by blunt divulsion and the polymer implanted. It is important to highlight that the divulsion was performed ventrally to the incision so that the skin suture did not interfere in the evaluations. Then, skin suture was performed in a simple interrupted pattern with nylon 0. Postoperative procedures consisted of cleaning the area using gauze and 0.9% saline solution and fly repellent ointment around the surgical wound once a day for 10 days. No analgesic and/or anti-inflammatory medication was provided during the experimental period. The stitches were removed on the 7th postoperative day. The same surgeon performed all surgical procedures.

### 4.5. Evaluation Methods

#### 4.5.1. Clinical Evaluation

Surgical wounds were evaluated regarding the integrity of the sutures and the presence of secretion. Physical examination quantified the heart rate (HR), respiratory rate (RR), rectal temperature (RT) as well as the intestinal motility, hydration status, apparent mucous color, and capillary filling time. Physical examination was performed before the commencement of the study and 6, 12, 24, 48, 72, 96, 120, 144, 168 h after implantation and then weekly up to 8 weeks, fortnightly up to 38 weeks, and 57 weeks. The same evaluator obtained all the measurements.

#### 4.5.2. Plasma Fibrinogen (PF)

To determine PF concentration, the chronometric technique described by Clauss (1957) [37] was used. Blood samples (3.6 mL) were collected in tubes containing 3.2% sodium citrate. Plasma was separated and frozen at −80 °C. After thawing, 200 µL aliquots of the samples were diluted in a buffer solution containing sodium barbital at a 1:10 ratio. Subsequently, 100 µL of thrombin (Fibrinogênio hemostasis, Labtest diagnóstica, Brazil) was added. Clot formation time was determined at 37 °C using a coagulometer (COAG 1000; Wama Diagnóstica, Brazil), which automatically converted the time obtained into fibrinogen concentration (mg/dL^−1^). PF was determined before the commencement of the study and 6, 12, 24, 48, 72, 96, 120, 144, 168 h after implantation and again at 2 and 3 weeks.

#### 4.5.3. Mechanical Nociceptive Threshold (MNT)

A commercial set of Von Frey Filaments (VFF) were used to assess the skin MNT (Touch-TestTM Sensory Evaluators, Stoelting Company, Wood Dale, IL, USA). Six filaments of sizes 5.07 to 6.65 were used, which represent applied forces ranging from 11.8 to 446.7 g, respectively. The filaments were applied perpendicularly to the horse’s skin until the nylon thread started to bend. Four applications, separated approximately 1 cm from each other, were performed around the implantation site, at intervals of 3 s. Initially, the thinnest filament was used and, when no aversive response was observed, the next filament was used until the animal showed an aversive reaction or the largest filament was used. An aversive reaction was defined as a movement of the tail, ears, or head, kicking, or stepping to the side. Simple motion reflexes upon first touching the filament on the skin were not accepted as an aversion response, and in these cases, the test was repeated after 10 s. Evaluations were performed before the commencement of the study and 6, 12, 24, 48, 72, 96, 120, 144, 168 h after implantation, then weekly until 8 weeks, and fortnightly until 38 weeks. The same operator performed all measurements, and these were made with the horse in a quadrupedal position in an area without movement restrictions. The values obtained were converted into force (g) according to the table provided by the manufacturer.

#### 4.5.4. Collection of Biopsy Specimens

The implants were removed along with surrounding tissue fragments. Two implants were removed after 24 weeks of implantation (PLA24 and PLA24F), one implant was removed at 28 weeks (PLA28), one implant at 34 weeks (PLA34), one at 38 weeks (PLA38), and the final implant was removed at 57 weeks (PLA57). As in the implantation procedure, the animal was properly sedated, and local infiltrative anesthesia was performed before the surgical removal of the fragment. One of the fragments removed after 24 weeks of implantation (PLA24F) was fixed in 10% buffered formalin for 24 h. The other implants and surrounding tissue fragments were fixed in 3.5% glutaraldehyde solution with PBS (pH 7.2) for 24–48 h and subsequently subjected to three washes using 5% glucose in 0.1 m sodium cacodylate buffer solution for 5 min. As we did not know whether fixation in glutaraldehyde would interfere with the histological evaluation and because this horse was used in the pilot study by Carvalho et al., 2020 [22], at 24 weeks two polymers were removed, one of which was fixed in formalin, and the other one in glutaraldehyde. After the slides were evaluated by an experienced pathologist, who confirmed that fixation in glutaraldehyde did not interfere with the histological quality, we proceeded to prepare the samples fixed in glutaraldehyde for SEM analysis. All materials were cut into two fragments, one for histopathological analysis and one for scanning electron microscopy (SEM), except for the PLA24F material, which was submitted to histopathological analysis only. Materials were stored in 70% alcohol until further use.

#### 4.5.5. Histopathological Analysis

For histopathologic evaluation, tissue samples were processed for paraffin embedding, and 5 µm sections were obtained and stained with hematoxylin and eosin. Tissue samples were evaluated in a light optic microscope by a pathologist with no knowledge about the implant’s removal time, thus eliminating bias. Lesions were classified semiquantitatively using the scores described by De Jong et al. (2005) [30], with some modifications. Briefly, classification included the characterization of the capsule involving the implant, the presence of an inflammatory infiltrate, cellular growth within the implant, and polymer’s phagocytosis. The intensity of each category was evaluated from mild to severe. The intensity of the inflammatory infiltrates, cellular growth within the implant, and polymer’s phagocytosis was evaluated as (1) minimal; (2) moderate; (3) marked; and (4) severe. In addition to scoring as proposed by De Jong et al. (2005) [30], the angioplasia in the capsule involving the polymers was also classified as (1) mild, when few small vases were observed within the fibrotic capsule; (2) moderate, when there were some moderately hyperemic vessels grouped around the capsule; and (3) marked, when diffusely hyperemic vessels were observed within the capsule.

#### 4.5.6. Scanning Electron Microscopy (SEM)

Morphological characterization of the materials was performed by SEM. Implant samples with surrounding tissue fragments underwent a dehydration process in an increasing alcohol solution and dried at a critical point. Samples that had the polymer removed from the obtained fragments were dried in an oven at 35 °C for 12 h. All samples were mounted on supports and sputtered with gold in a vacuum. The material surfaces were analyzed in a scanning electron microscope (Zeiss EVO 10, Zeiss, Oberkochen, Germany) operated at 10–15 kV. Images were examined with Fiji ImageJ 1.50i software, and the average pore diameter was determined manually from at least 300 pores. For non-circular pores, the greatest distance was considered.

### 4.6. Statistical Analysis

All variables were checked for normal distribution using the Shapiro-Wilk test. To evaluate the MNT, the six implants were considered as repetitions (*n* = 6) for the same animal. MNT was subjected to analysis of variance (ANOVA) for repeated measurements in unilateral time, followed by Tukey’s post-hoc test. Pore diameter evaluated by SEM images were submitted to the Kruskal–Wallis H-test for one-way analysis of variance (ANOVA), followed by the Dunn’s post-hoc test. All statistical analyzes were performed using Sigma-Plot software, version 12.0, with a significance level of *p* < 0.05.

## 5. Conclusions

Over time the polymer changed its morphology, indicating biocompatible degradation. The kinetics of PLA biodegradation revealed herein reinforce PLA’s potential for clinical use and may contribute to future research efforts to enable its use in the biomedical industry, including equine medicine.

## Figures and Tables

**Table 1:** Score classification of the histopathological lesions in the implant samples at each surgical removal time of a horse implanted with six polymers of poly(lactic acid) (PLA). Intensity of lesions was classified as mild (1), moderate (2), marked (3), and severe (4).

Polymer	Capsule Characterization	Infiltrate/ Inflammation	Cellular Growth	Phagocytosis	Angioplasia
PLA24	1	1	1	2	1
PLA24F	2	1	3	1	2
PLA28	2	2	2	1	1
PLA34	2	2	3	1	2
PLA38	3	3	4	3	2
PLA57	Ns	Ns	Ns	Ns	Ns

PLA24: 24 weeks following implantation; PLA24F: 24 weeks following implantation, formalin-fixed; PLA28: 28 weeks following implantation PLA34: 34 weeks following implantation; PLA38: 38 weeks following implantation; PLA57: 57 weeks following implantation. Ns: not scored.

**Table 2:** Pore median diameter of PLA implanted subcutaneously in one horse and removed at different times.

Polymer	Pore Median Diameter (μm) (IQR)
PLA24	0.477 (0.354–0.624)
PLA28	1.251 (0.815–1.846) *
PLA34	0.751 (0.487–1.353) *
PLA38	1.254 (0.914–1.618) *

PLA24: 24 weeks following implantation; PLA28: 28 weeks following implantation; PLA34: 34 weeks following implantation; PLA38: 38 weeks following implantation. * Indicates larger pore diameter. IQR, interquartile range.

## Data Availability

The raw/processed data required to reproduce these findings are available from the authors upon request.

## Supplementary Materials

The following are available online, Figure S1: Physiological variables of a horse submitted to the implantation of six poly(lactic acid) (PLA) samples. (A) Heart rate (HR), (B) respiratory rate (RR) and (C) rectal temperature (RT). Figure S2: Plasma fibrinogen concentration (mg/dL) of a horse submitted to the implantation of six polymers of poly(lactic acid) (PLA).
