# Supplementary material for: Long-Term Evaluation of Poly(lactic acid) (PLA) Implants in a Horse: An Experimental Pilot Study
Source: Molecules. 2021 Nov 29;26(23):7224. doi: 10.3390/molecules26237224 (PMC8658935; doi:10.3390/molecules26237224)
Supplement: Supplementary file 1 [file molecules-26-07224-s001.zip › molecules-1444465-supplementary.pdf]

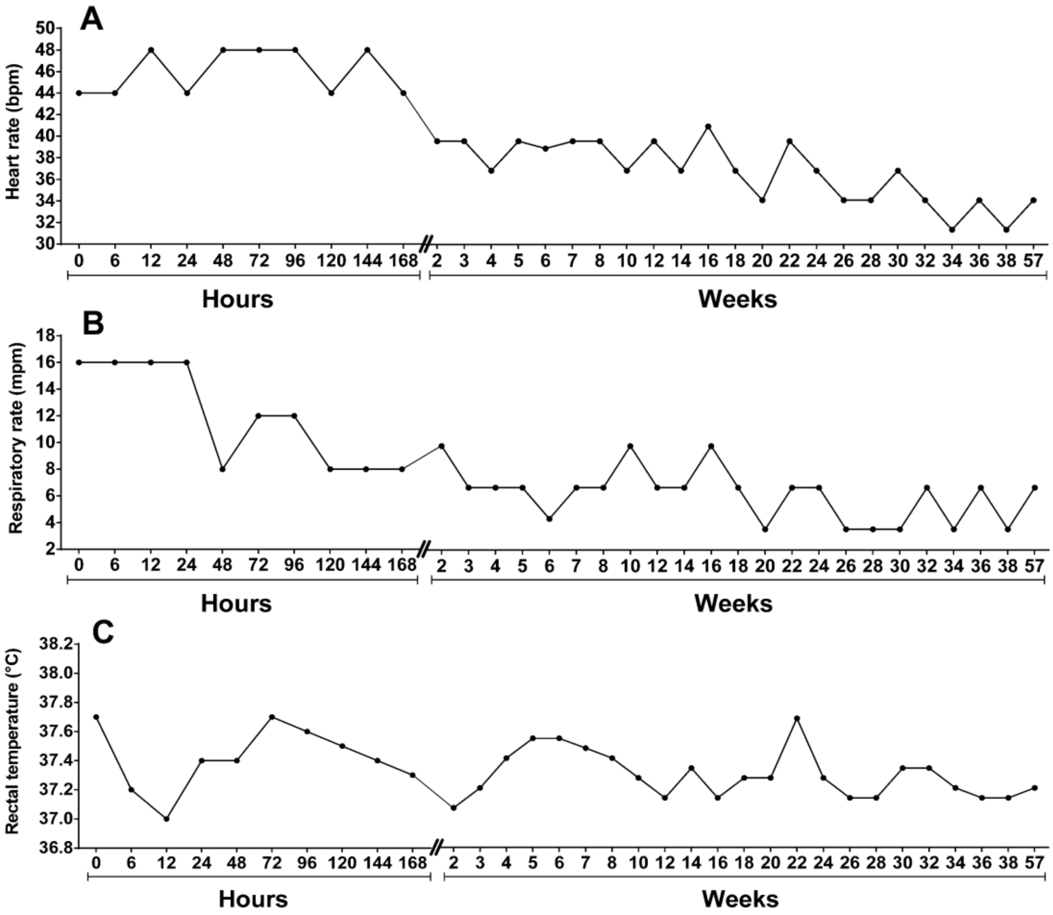

Figure S1. Physiological variables of a horse submitted to the implantation of six poly(lactic acid) (PLA) samples. (**A**) Heart rate (HR), (**B**) respiratory rate (RR) and (**C**) rectal temperature (RT).

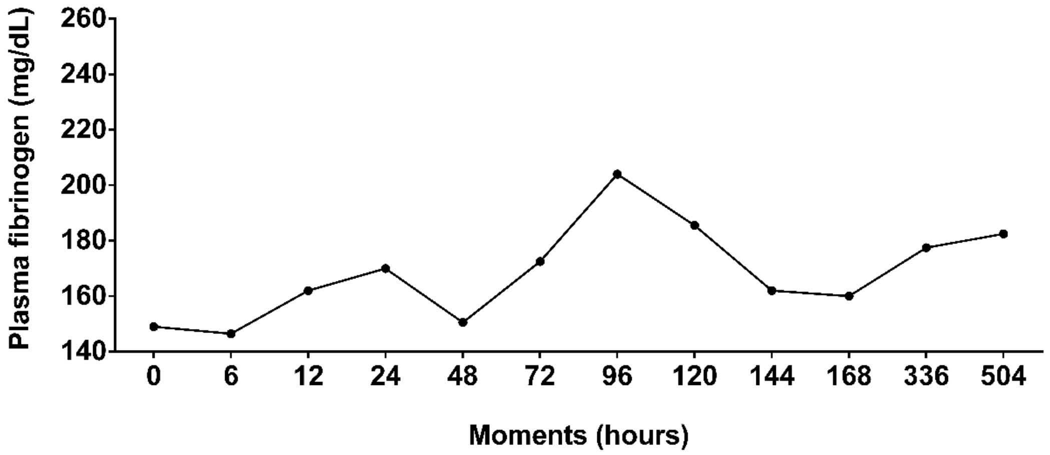

Figure S2. Plasma fibrinogen concentration (mg/dL) of a horse submitted to the implantation of six polymers of poly(lactic acid) (PLA).
